# Chemical Degradation of Waste Polyethylene Terephthalate and Its Application in Developing Asphalt Additives

**DOI:** 10.3390/ma19194105

**Published:** 2026-09-25

**Authors:** Jingzhuo Zhao, Yong Huang, Kai Su, Xingzhen Zang, Hui Wang, Rui Dong

**Affiliations:** 1 School of Ecology and Environment, Xinjiang University, Urumqi 830046, China; 2 Gansu Province Transportation Planning Survey & Design Institute Co., Ltd., Lanzhou 730030, China

**Keywords:** polyethylene terephthalate, solid waste resource utilization, chemical degradation, asphalt additive

## Abstract

To address the low resource-recovery efficiency of waste polyethylene terephthalate (PET) and the shortage of high-performance eco-friendly asphalt modifiers, PET-derived functional additives were fabricated in this work via zinc-acetate-catalyzed alcoholysis of waste PET followed by repolymerization of the obtained degradation fragments. Owing to the diverse origins of waste PET feedstock, noticeable fluctuations in molecular-weight parameters are commonly encountered. Nevertheless, waste PET can be effectively depolymerized into relatively low-molecular-weight derivatives under the proposed catalytic alcoholysis conditions. This study systematically investigated the effects of different PET degradation pathways on the molecular architecture, molecular-weight distribution, and thermal stability of as-prepared PET-derived asphalt additives. It further uncovers the intrinsic structure–performance relationships between molecular characteristics and critical asphalt properties, including rheological behavior, interfacial adhesion, high-temperature rutting resistance, and low-temperature elastic recovery. Homogeneous alcoholysis conducted at 220 °C enables efficient depolymerization of waste PET to yield derivatives with tailorable molecular weights. The resultant amphiphilic alcoholysis products can reduce asphalt viscosity and ameliorate water stability, rendering them promising warm-mix anti-stripping additives. By contrast, repolymerized PET derivatives are capable of constructing cross-linked networks within asphalt matrix, which substantially boost the high-temperature modulus and fatigue resistance of modified asphalt. Distinct from conventional physical blending approaches that are plagued by inferior interfacial compatibility and marginal performance improvement, this chemical modification strategy affords PET-based additives with tunable functions for different engineering scenarios. These findings provide technical support for high-value recycling of waste PET and the development of sustainable low-carbon asphalt materials.

## 1. Introduction

Polyethylene terephthalate (PET) is one of the most extensively utilized synthetic polymers worldwide due to its superior mechanical properties, low water absorption, excellent chemical inertness, and cost-effectiveness, which has been widely applied in food packaging, textiles, thin films, adhesives, and engineering plastics [1,2,3]. Figure 1 displays the Fourier transform infrared (FTIR) spectra of five real-world post-consumer waste PET fragments (PET1–PET5) collected from municipal solid waste. These samples are only used to characterize the general molecular features of real-life waste PET, and none of these five fragments were adopted for the formal degradation experiments. The formal degradation experiments in this study adopt commercially purchased recycled PET pellets (detailed parameters in Section 2.1). The FTIR results confirm that real-world waste PET shares nearly identical main molecular skeleton with our experimental PET pellets, which demonstrates that our pellet feedstock is representative for practical waste PET recycling. Conventional disposal strategies for waste PET, including direct recycling, landfilling, and incineration, suffer from low resource-recovery efficiency, low added economic value, and severe secondary environmental contamination. Such limitations fail to comply with the global dual-carbon strategic goals [4,5,6]. To facilitate the resource utilization and high-value upgrading of waste plastics, numerous countries have promulgated targeted policies to encourage the innovative collaborative disposal of solid waste in transportation engineering, building materials, and environmental protection industries [7,8,9]. Under this strategic context, the exploration of efficient degradation and high-value utilization technologies for recycled PET is of profound social, economic, and environmental significance and holds promising engineering application potential.

The comprehensive service performance of asphalt pavement is highly dependent on functional modifiers. The incorporation of recycled PET into asphalt modification provides a feasible approach for large-scale waste plastic consumption. Meanwhile, recycled PET can ameliorate the high-temperature rutting resistance, low-temperature cracking resistance, water stability, and fatigue durability of asphalt, conforming to the development orientation of green transportation and low-carbon road infrastructure construction [10,11,12,13,14]. Nevertheless, previous studies have proven that the direct incorporation of pristine PET particles into asphalt presents inherent technical drawbacks. The poor interfacial compatibility, uneven particle dispersion, and severe agglomeration behavior severely restrict the modification efficiency, resulting in limited performance improvement and unsatisfactory engineering applicability [15,16,17].

Compared with physical blending, chemically degraded PET derivatives enriched with polar functional groups (e.g., ester, amide, and hydroxyl groups) can effectively optimize the interfacial compatibility and functional adaptability between modifiers and asphalt matrices, thus improving the rheological behaviors and pavement service performance of asphalt [18,19,20]. Accordingly, chemical modification has become a critical technical route to break through the bottlenecks of conventional physical modification, emerging as a research hotspot for the high-value recycling of waste PET. For instance, Li et al. synthesized amphiphilic oligomeric additives via glycolysis of waste PET. Combined with molecular dynamics simulations and experimental verification, the synthesized additives were confirmed to effectively enhance the workability, interfacial adhesion, and water stability of asphalt binders [21]. Zou et al. further verified that PET aminolysis derivatives could serve as high-efficiency asphalt modifiers, which conferred superior moisture damage resistance and optimized viscoelastic and rutting resistance properties to asphalt [22]. Industrially, Kao Corporation has successfully commercialized the PET-based modifier NEWTLAC 5000 [23]. This product can prominently strengthen the adhesion between asphalt and mineral aggregates and has been practically applied in engineering projects. Nevertheless, several research gaps still exist in the current investigations of chemically degraded PET for asphalt modification. First, most existing studies focus on a single degradation pathway, whereas systematic comparative analyses of degradation modes, functional group characteristics, and molecular structures are insufficient. Second, the structure-activity relationship between PET derivatives and asphalt performance remains ambiguous, and the modification mechanism is merely explained at the macroscopic phenomenological level. Furthermore, theoretical guidance concerning the thermal stability of degradation products, optimal dosage criteria, and the synergistic optimization of high- and low-temperature performance is still lacking.

Against the above research background, waste PET was selected as the raw material to fabricate asphalt additives with diverse functional groups and molecular structures via ethylene glycol (EG)/polyethylene glycol (PEG) alcoholysis combined with secondary polymerization. Multiple characterization techniques, including Fourier transform infrared spectroscopy (FTIR), gel permeation chromatography (GPC), and thermogravimetry/differential thermal analysis (TG/DTA), were employed to characterize the molecular structure and thermal stability of modified PET products. The rheological properties, interfacial adhesion, high-temperature stability, and low-temperature ductility of neat asphalt and SBS-modified asphalt were comprehensively evaluated through a series of tests, involving Brookfield rotational viscosity, softening point measurement, dynamic shear rheometer (DSR), contact angle detection, adhesion test, and elastic recovery test. The intrinsic correlations among molecular structure, interfacial interaction, and pavement performance were clarified. This study not only provides a reliable theoretical basis for the high-value recycling of waste PET and an innovative technical scheme for the preparation of green low-carbon asphalt additives, but also promotes the resource utilization of solid waste and the sustainable development of the transportation industry.

## 2. Experimental

### 2.1. Raw Materials

Recycled PET pellets (commercially available, particle size: 2–3 mm, number-average molecular weight: 15,000–35,000 g/mol, weight-average molecular weight: 30,000–65,000 g/mol, polydispersity index: 1.5–2.0, supplied by Lanzhou Jindi Plastic Products Co., Ltd., Lanzhou, China); zinc acetate dihydrate (analytical-grade, used as alcoholysis catalyst, 2 wt% relative to the mass of PET, supplied by Shanghai Macklin Biochemical Co., Ltd., Shanghai, China); ethylene glycol (EG, analytical reagent, supplied by Shanghai Macklin Biochemical Co., Ltd., Shanghai, China); polyethylene glycol (PEG-200/400/600, analytical reagent, supplied by Shanghai Macklin Biochemical Co., Ltd., Shanghai, China); trimellitic acid (TMA, analytical reagent, supplied by Shanghai Macklin Biochemical Co., Ltd., Shanghai, China); malonic acid (MA, analytical reagent, supplied by Shanghai Macklin Biochemical Co., Ltd., Shanghai, China); succinic acid (SAA, analytical reagent, supplied by Shanghai Macklin Biochemical Co., Ltd., Shanghai, China); glutaric anhydride (GAH, analytical reagent, supplied by Shanghai Macklin Biochemical Co., Ltd., Shanghai, China); glycerol (GLY, analytical reagent, supplied by Shanghai Macklin Biochemical Co., Ltd., Shanghai, China); tetrabutyl titanate (TBT, analytical reagent, supplied by Shanghai Macklin Biochemical Co., Ltd., Shanghai, China); xylene (analytical reagent, supplied by Shanghai Macklin Biochemical Co., Ltd., Shanghai, China); base asphalt (ZH90, supplied by PetroChina Liaohe Petrochemical Company, Panjin, China); 4.5 wt% SBS-modified asphalt (styrene unit mass fraction: 30 wt%, number-average molecular weight: 80,000–120,000 g/mol, weight-average molecular weight: 120,000 g/mol, polydispersity index: 1.3–1.7, supplied by PetroChina Liaohe Petrochemical Company, Panjin, China). All reagents were used as received without further purification.

### 2.2. Preparation of PET-Derived Asphalt Additives

#### 2.2.1. Alcoholysis of PET

As illustrated in Figure 2 and Figure 3, alcoholysis reaction schemes of waste PET with ethylene glycol (EG) and polyethylene glycol (PEG). We used EG and PEG with different molecular weights as alcoholysis agents. PET and alcoholysis agent were added into a three-necked flask at a mass ratio (m_PET_: m_malcoholysis agent_) from 2:1 to 6:1. Zinc acetate dihydrate (2 wt% relative to PET mass) was added as the alcoholysis catalyst. Nitrogen was introduced as a protective atmosphere. Under atmospheric pressure and mechanical stirring at 300 r/min, the reaction system was heated to 160 °C or 220 °C for isothermal alcoholysis for 3 h, then cooled to room temperature. Residual alcoholysis agent was removed by washing with deionized water. After silk filtration and vacuum drying to constant weight, PET alcoholysis products under different process conditions were obtained.

#### 2.2.2. Secondary Polymerization

As illustrated in Figure 4 and Figure 5, the secondary polymerization of degraded PET was performed taking glutaric anhydride (GAH) and malonic acid (MA) as examples, diacids (SAA, GAH, MA), triacid (TMA) and trialcohol (GLY) were selected as comonomers to regulate the spatial structure of products by controlling monomer functionality. Before the reaction, degradation products were dried in a vacuum drying oven at 80 °C for 2 h to remove residual moisture. Degradation products, comonomers and tetrabutyl titanate (TBT, 0.03 wt% of the total mass of reactants, used as polycondensation catalyst) were added into a three-necked flask at a preset molar ratio (prior to secondary polymerization, the hydroxyl value of PET alcoholysis oligomers was measured by acetic-anhydride titration, to quantify the molar amount of terminal hydroxyl groups. The feeding molar ratio between measured terminal-OH groups (from degradation products) and carboxyl/hydroxyl functional groups of comonomers was set to 1:1.05 accordingly. An appropriate amount of xylene was introduced as the reflux solvent, and nitrogen protection was implemented throughout the reaction process. Under atmospheric pressure and stirring at 300 r/min, the reaction system was slowly heated to 180 °C and kept isothermal for 1 h to ensure sufficient mixing and preliminary esterification. Then the temperature was raised to 190–200 °C for further isothermal reaction for 3 h to complete esterification and polycondensation. When the system viscosity reached 500–800 mPa·s (25 °C), heating and stirring were stopped. The crude product was poured out and cooled to room temperature, followed by deionized-water washing to remove residual hydroxyl-containing small-molecule impurities. Subsequently, the purified product was dried in a vacuum drying oven at 80 °C to constant weight for subsequent asphalt modification and performance characterization.

### 2.3. Characterization

Molecular structure and thermal properties: FTIR spectra (NICOLET 6700 spectrometer, Thermo Fisher Scientific, Madison, WI, USA); GPC measurements (SHIMADZU RID-20A system, Shimadzu Corporation, Kyoto, Japan); TG/DTA analyses (SHIMADZU DTG-60AH, Shimadzu Corporation, Kyoto, Japan).

Asphalt performance: Brookfield rotational viscosity (BROOKFIELD DV2T, AMETEK Brookfield, Middleboro, MA, USA); Softening point (CONTROLS 81-PV014, CONTROLS S.p.A., Liscate, Italy); DSR tests (DISCOVERY HR10 rheometer, TA Instruments, New Castle, DE, USA); Contact angle (Biolin Scientific Oy, Biolin Scientific Oy, Espoo, Finland); Elastic recovery rate (CONTROLSGROUP 81-PV10020, CONTROLS S.p.A., Liscate, Italy); Asphalt-aggregate adhesion was evaluated according to standard procedures.

Two types of PET-derived additives were prepared in this study, including alcoholysis-series additives and repolymerization-series additives. To match their functional characteristics, alcoholysis-series additives were blended with 4.5 wt% SBS-modified asphalt, while repolymerization-series additives were mixed with ZH90 base asphalt. The additive dosages were 1%, 2%, 3%, 4%, and 5% by mass of asphalt binder.

Alcoholysis products act as warm-mix anti-stripping agents and are intended to improve the workability of SBS-modified asphalt, which normally suffers from high viscosity. Repolymerized PET derivatives form cross-linked networks inside asphalt and are designed to enhance the high-temperature modulus and anti-rutting performance of neat base asphalt. Detailed mixing proportions are summarized in Table 1.

## 3. Results and Discussion

### 3.1. Structure and Thermal Stability of Degradation Products

#### 3.1.1. Preparation of PET Alcoholysis Products

All alcoholysis reactions described in this section were performed under catalysis by 2 wt% zinc acetate dihydrate. As EG was used as the alcoholysis agent, the main product of recycled PET under sufficient degradation was bis(2-hydroxyethyl) terephthalate (BHET). The alcoholysis process proceeds via an SN2-type nucleophilic substitution mechanism. Hydroxyl groups from EG attack the ester carbonyls on PET chains, promoting main-chain cleavage and ester bond dissociation, forming hydroxyl-terminated oligomers and the monomeric ester product. Figure 6 shows molecular-weight distributions after alcoholysis at 160 °C and 220 °C with different PET/EG ratios.

Reaction phase and mass transfer kinetics strongly influence degradation. At 160 °C, PET cannot be fully dissolved; the system is a heterogeneous solid–liquid two-phase mixture. EG acts only on the PET surface and cannot penetrate the internal segments, resulting in high interfacial mass transfer resistance. Increasing EG dosage does not improve mixing uniformity or reaction efficiency, leading to a wide molecular-weight distribution and uncontrollable product structure. At 220 °C, PET is completely dissolved in EG and forms a homogeneous transparent system with EG, allowing full molecular-scale contact and diffusion. Nucleophilic substitution occurs throughout the system, accelerating ester exchange kinetics. With increased EG dosage, the hydroxyl concentration rises, further promoting chain cleavage and end-capping, increasing the proportion of small molecules and narrowing the molecular-weight distribution.

Thus, temperature not only provides energy for the ester exchange reaction but also promotes the dissolution of PET in diol solvent to form a homogeneous reaction system, greatly reducing diffusion resistance and enhancing reaction efficiency.

When PEG is used to degrade PET, the molecular-weight of products correlates positively with that of PEG. PEG molecules with hydroxyl groups at both ends can nucleophilically attack ester bonds of PET similarly to EG alcoholysis, but they achieve insertion-type co-alcoholysis rather than chain-scission depolymerization. The larger the molecular weight of PEG, the longer the molecular chain, the more prominent the steric hindrance and segment flexibility, and the easier it is to combine with PET segments in a whole-chain embedding manner, forming PET-PEG-PET block oligomers-different from EG alcoholysis that completely degrades PET into small molecular monomers. Figure 7 shows molecular weight normalization and FTIR of PET after alcoholysis with PEG-200/400/600.

Under the same temperature, dosage ratio, and reaction time, PEG-200 (short chain, fast diffusion rate, high hydroxyl density) is more likely to cleave PET main chains multiple times, generating lower-molecular-weight degradation products. PEG-400/600 (long segments) can complete end-capping and segment coupling with a single molecule, making the degradation products retain longer rigid segments. The molecular-weight of final products increased gradually with PEG molecular weight. Meanwhile, PEG molecular weight affects product distribution by changing compatibility and reaction kinetics. High-molecular-weight PEG has better compatibility with PET dissolved in diol solvent and react uniformly in a liquid homogeneous system, leading to a more regular molecular weight distribution. Low-molecular-weight PEG has strong volatility, easily causing uneven local reaction and slightly wider product distribution. Therefore, PEGs with different molecular weights enable precise regulation of the molecular weight of degradation products, providing a solid foundation for tailoring asphalt modifications to diverse requirements.

As shown by the TGA-DTA curves of PEG-200, PEG-400 and PEG-600 alcoholysis products in Figure 8, the thermal decomposition of all three products proceeds in two main stages. The first stage occurs around 150–300 °C, corresponding to the decomposition of low-molecular-weight fractions and terminal hydroxyl groups. The second stage occurs between 300 °C and 450 °C, corresponding to the cleavage of ester bonds and polyether main chains. The initial thermal decomposition temperature of the PEG-200 alcoholysis product is approximately 165 °C. By contrast, the values for PEG-400 and PEG-600 products exceed 180 °C, demonstrating that increasing the molecular weight of PEG effectively enhances the thermal stability of the alcoholysis products. At 800 °C, the residual mass rates of the three products are approximately 8.2%, 12.5% and 15.8%, respectively. The increase in residual mass with PEG molecular weight indicates that higher-molecular-weight PEG derivatives contain more thermally stable aromatic structures, which is consistent with the higher thermal decomposition temperature.

The DTA curves show a distinct endothermic peak around 200–250 °C, which corresponds to the first-stage thermal decomposition. As the molecular weight of PEG increases, this endothermic peak shifts toward higher temperatures and its intensity gradually weakens. A second weaker endothermic peak appears around 380–420 °C, corresponding to the cleavage of the main chain. The peak temperature of PEG-600 product is about 30–40 °C higher than that of PEG-200 product, further confirming the improved thermal resistance.

The initial decomposition temperature of the PEG-200 alcoholysis product falls within, or even below, the conventional asphalt mixing temperature range of 160–180 °C. If PEG-200 alcoholysis product is adopted as asphalt modifier, obvious thermal degradation will occur during high-temperature asphalt mixing. This thermal degradation will produce volatile low-molecular polyether fragments, release unpleasant odor, destroy the amphiphilic molecular structure of modifier, and further lead to deterioration of viscosity-reduction and anti-stripping performance. Therefore, PEG-200 alcoholysis product is not suitable for practical engineering applications. In view of this, PEG-400 and PEG-600 alcoholysis products are preferable for practical engineering applications. Their initial decomposition temperatures are higher than 180 °C, so the molecular structure remains resistant to thermal decomposition throughout asphalt mixing, storage and construction at high temperatures. Accordingly, these additives can stably maintain their modification performance and are more suitable for asphalt pavement under high-temperature service conditions.

Such a trend originates from the combined effects of chain segment length, van der Waals forces and molecular thermal motion. PEG with lower molecular weight possesses a higher proportion of terminal hydroxyl groups and shorter chain segments, which reduces the activation energy for molecular motion. Upon heating, the product is susceptible to terminal dehydration, chain disentanglement and ester bond cleavage, leading to a low onset decomposition temperature. In contrast, higher-molecular-weight PEG provides longer molecular chains and stronger van der Waals interactions, improving thermal stability and delaying thermal cracking. The introduction of long flexible polyether segments further elevates the decomposition temperature of the derivatives.

#### 3.1.2. Preparation of PET Secondary Polymerization Products

Secondary polymerization enables the rational molecular structure design and modulation of PET degradation derivatives. Products with one-dimensional linear structures and three-dimensional network architectures can be controllably synthesized through stepwise polycondensation. During the polycondensation reaction, terminal hydroxyl groups of PET degradants react with carboxylic acid monomers via esterification and dehydration, thereby realizing molecular chain growth and structural reconstruction. When difunctional diols and diacids are adopted as raw materials, molecular chains extend in a single dimension to form linear structures. Such linear architectures possess favorable segment flexibility but exhibit weak intermolecular interactions and inferior structural stability. By contrast, the introduction of trifunctional monomers (e.g., TMA and GLY) induces multi-point branching and intermolecular cross-linking during polycondensation. The spatially interconnected molecular chains construct a compact three-dimensional cross-linked network, which significantly enhances thermal stability, structural rigidity, and deformation resistance. Consequently, cross-linked products effectively resist molecular segment cleavage and flow deformation under high-temperature conditions. Figure 9 presents the normalized molecular-weight distributions of secondary polymerization products derived from different alcoholysis routes, including EG, MA, SAA, GAH, and TMA.

The alcoholysis products display relatively low number-average molecular weight (Mn) and weight-average molecular weight (Mw), accompanied by a narrow unimodal peak. With the increase in PEG molecular weight (PEG-200, PEG-400, PEG-600), both Mn and Mw of alcoholysis products gradually rise, while the peak position shifts toward higher molecular weight region. The polydispersity index (PDI) of alcoholysis products remains at a relatively low level, indicating relatively uniform molecular chain length after alcoholysis depolymerization.

In contrast, the repolymerization product presents an obviously shifted GPC peak at much higher molecular-weight, with significantly increased Mn and Mw compared with all alcoholysis samples. Meanwhile, its PDI becomes slightly broader, which originates from the random chain growth reaction during secondary polymerization. The broader molecular weight distribution implies that polymer chains with various lengths are formed, consistent with the construction of cross-linked network precursors.

The GPC results further verify the structural difference between two types of PET-derived additives. Alcoholysis breaks the long PET macromolecular chains into short-chain fragments with tunable molecular weight, which endows the product with amphiphilic characteristics and lubricating effect for asphalt. Secondary polymerization reconnects these short fragments into longer macromolecular chains, laying the structural foundation for forming cross-linked network in asphalt matrix and enhancing high-temperature modulus and rutting resistance.

The TGA-DTA curves of the synthesized secondary polymerization products are displayed in Figure 10. The linear MA product exhibits an initial thermal decomposition temperature of 152 °C, while all cross-linked products (SAA, GAH, and TMA) show an elevated initial decomposition temperature above 190 °C. This distinction verifies the superior thermal stability of three-dimensional network-structured derivatives. Such performance discrepancy originates from inherent structural differences. Linear products are fabricated through the polycondensation of difunctional monomers, forming one-dimensional molecular chains without chemical cross-linking. Molecular segments are merely bound by weak van der Waals forces, lacking stable cross-linking nodes. Upon heating, these structures are susceptible to chain disentanglement, ester bond cleavage, and small-molecule volatilization, resulting in a low initial decomposition temperature and rapid mass loss.

In comparison, SAA, GAH, and TMA-modified products form dense three-dimensional network frameworks with covalently interconnected molecular chains. This unique structure increases the activation energy for molecular thermal motion and restricts chain slippage and thermal degradation at high temperatures. Furthermore, the incorporation of aromatic TMA further strengthens the rigidity and thermal stability of the molecular skeleton. The prominent mass loss of cross-linked network products only occurs at temperatures exceeding 190 °C. Such satisfactory thermal stability ensures structural integrity during asphalt construction, eliminating performance deterioration caused by thermal degradation. Therefore, these three-dimensional cross-linked products are promising candidates for the fabrication of high-performance asphalt modifiers.

### 3.2. Effects of Degradation Products on Asphalt Performance

#### 3.2.1. Alcoholysis Products

All performance tests for alcoholysis-series additives in this subsection were performed on 4.5 wt% SBS-modified asphalt. As depicted in Figure 11, the incorporation of PEG alcoholysis products exhibits a negligible regular effect on the softening point of modified asphalt.

As shown in Figure 12, the effect of PEG alcoholysis products on asphalt viscosity at 135 °C, 150 °C and 180 °C exhibits obvious condition-dependence. The viscosity decreases under certain dosages and PEG molecular-weight conditions, slightly increases under others, and shows negligible variation in some cases. Notably, significant viscosity reduction was not achieved across all test scenarios. Brookfield rotational viscosity tests in this study were performed at a constant rotor speed of 20 s^−1^. Such irregular viscosity variation implies a potential risk of local phase separation within modified-asphalt system (this remains a speculation derived from macroscopic rheological observations; direct microscopic evidence such as fluorescence microscopy or AFM is not available in current work to confirm such phase morphology). The viscosity reduction under partial dosages greatly facilitates high-temperature construction workability, demonstrating favorable warm-mix characteristics. Moreover, the elevated dosage of additives slightly declines the high-temperature rheological indices with an overall downward trend.

PEG alcoholysis molecules simultaneously contain rigid benzene ring segments, polar ester units, and flexible polyether chains. The aromatic segments possess superior compatibility with aromatic fractions and resins within asphalt matrices. Nevertheless, polar polyether segments exhibit relatively high polarity and limited compatibility with non-polar asphalt binder. It is hypothesized that modifier molecules show amphiphilic nature: benzene-ester moieties are miscible with asphalt continuous phase, whereas polar polyether segments tend to be immiscible with asphalt matrix, which may lead to formation of polyether-rich dispersed droplets. This hypothesis is analogous to the phase-separation phenomenon reported for bio-oil modified asphalt, where part of additive components are miscible with heavy-hydrocarbon asphalt matrix while polar fractions separate into discrete droplets [24]. It should be emphasized that this phase-separation-droplet hypothesis has not been directly verified by microscopic characterizations in the present study. The polar polyether chains exert an evident lubricating effect, which can intercalate into the layered structure of resins and asphaltenes. This behavior weakens intermolecular π-π conjugation and hydrogen bonding, thereby reducing asphalt viscosity and achieving desirable warm-mix performance.

Figure 13 presents the adhesion force and contact angle variations in asphalt modified by PEG alcoholysis products. The amphiphilic molecular structure enables the products to preferentially adsorb at the asphalt-aggregate interface. Specifically, the internal ester groups and ether bonds can form hydrogen bonds or chemical interactions with hydroxyl and siloxy groups on aggregate surfaces. Such interfacial interactions strengthen asphalt-aggregate adhesion and endow asphalt with anti-stripping capability. The increased adhesion force and decreased contact angle effectively suppress water-induced peeling behavior, further improving the moisture stability of asphalt mixtures.

Figure 14a illustrates the combined influences of three variables, i.e., the molecular weight of PEG (M.W. of PEG), modifier dosage, and test temperature, on the rutting factor (G*/sinδ) of 4.5 wt% SBS-modified asphalt.

The three axes are defined as follows: Vertical axis (*Z*-axis): Molecular weight of PEG (M.W. of PEG), including three levels of 200, 400 and 600; *X*-axis: Dosage of PEG alcoholysis products, ranging from 0.5% to 2.5%; *Y*-axis: Test temperature, ranging from 55 °C to 85 °C. The color bar on the right represents the magnitude of rutting factor G*/sinδ(kPa). Each colored point corresponds to the measured rutting factor under a specific combination of PEG molecular weight, modifier dosage and temperature, connected by lines to show the trend.

Overall, the rutting factor decreases as temperature rises at fixed PEG molecular weight and dosage. With increasing modifier dosage, the rutting factor presents variable trends dependent on PEG molecular weight. For a given dosage and temperature, higher PEG molecular weight of the alcoholysis product generally yields higher rutting factor. A higher rutting factor means better high-temperature rutting resistance of asphalt.

Figure 14b demonstrates the combined effects of three variables, namely PEG molecular weight (M.W. of PEG), modifier dosage and test temperature, on the fatigue factor (G∗⋅sinδ) of 4.5 wt% SBS-modified asphalt.

Definitions of three axes: *Z*-axis (vertical): Molecular weight of PEG (M.W. of PEG), including three levels: 200, 400 and 600; *X*-axis: Dosage of PEG alcoholysis products, ranging from 0.5% to 2.5%; *Y*-axis: Test temperature, ranging from 55 °C to 85 °C. The color bar on the right denotes the magnitude of fatigue factor G∗⋅sinδ (kPa). Each colored dot represents the fatigue factor measured under a specific combination of PEG molecular weight, modifier dosage and temperature, and dots are connected by lines to visualize the changing trend.

In general, at fixed PEG molecular weight and dosage, the fatigue factor decreases as test temperature increases. Under the same dosage and temperature, higher molecular weight of PEG alcoholysis product yields a larger fatigue factor. The variation trend of fatigue factor with modifier dosage depends on the PEG molecular weight. A smaller fatigue factor indicates better fatigue cracking resistance for asphalt binder.

As shown in Figure 15a,b, after incorporating PEG alcoholysis products, the rutting factor decreases moderately, which suggests a slight degradation in high-temperature anti-rutting capacity. Meanwhile, the reduced fatigue factor demonstrates improved anti-fatigue-cracking performance of modified asphalt. This divergent performance originates from the introduced flexible polyether segments. The polyether segments enhance the overall flexibility and fluidity of asphalt matrix, which weakens high-temperature deformation resistance to a certain extent, yet facilitates stress dissipation and delays crack initiation under cyclic loading.

#### 3.2.2. Secondary Polymerization Products

All performance tests for repolymerization-series additives in this subsection were performed on ZH90 base asphalt. After modifying ZH90 base asphalt with secondary polymerization products, the system viscosity increases slightly, but the viscosity increase in the high-temperature zone is significantly lower than that at ambient temperature. The softening point decreases slightly, and the dosage effect shows no obvious regularity. With increasing dosage of secondary polymerization products, the rutting factor and fatigue factor of asphalt increase continuously. Three-dimensional network products significantly improve the high-temperature rutting resistance of asphalt and also improve the elastic recovery rate at low temperatures, achieving simultaneous optimization of high-temperature deformation resistance and low-temperature cracking resistance, making them excellent high-modulus or high-viscosity modifiers.

Secondary polymerization products use PET degradation products as a segment matrix and form linear or three-dimensional network molecular structures after polycondensation of multifunctional monomers, creating a complex interaction system with asphalt. Long-chain and branched structures increase internal friction of the system, leading to a slight viscosity increase. However, fluidity of asphalt is greatly enhanced at high temperatures, and product molecules exist in a dispersed state, not significantly affecting construction workability [25].

As presented in Figure 15, which illustrates the effect of secondary polymerization products on the softening point and viscosity of asphalt, the softening point of modified asphalt shows no obvious regularity with the change in additive dosage. Such irregular variation in fundamental property (softening point) versus modifier dosage generally indicates potential phase separation and insufficient compatibility inside modified-asphalt system. Our interpretation that this phenomenon arises from counter-balancing plasticizing effect of linear fractions and structural reinforcement effect of 3D-network fractions remains mechanistic speculation. Direct experimental evidence from micro-morphology characterizations (e.g., AFM, fluorescence microscopy) or multi-mode rheological measurements is still lacking and needs further validation in future work. This phenomenon is closely related to the amphiphilic structure of the secondary polymerization product molecules: linear products exert a certain plasticizing effect, which slightly lowers the asphalt softening point; in contrast, three-dimensional network products provide structural support for the asphalt system, offsetting part of the softening point decline caused by linear products. Under the combined action of these two opposite effects, there is no monotonic relationship between the dosage of secondary polymerization products and the asphalt softening point.

G* refers to the complex shear modulus, a core parameter obtained from the dynamic shear rheometer (DSR).

(1) G* = τ/γ

τ: shear stress;

γ: shear strain.

Complex form:

(2) G* = G′ + iG′′

G′ (storage modulus, elastic component) represents recoverable elastic deformation and denotes the elastic stiffness of the material;

G′′ (loss modulus, viscous component) represents irreversible viscous flow deformation and characterizes viscous dissipation.

|G*| is the magnitude of the complex modulus, which describes the overall stiffness of asphalt binders against shear deformation under oscillatory shear loading.

A larger G* indicates higher asphalt stiffness, stronger resistance to shear deformation, and better potential for high-temperature rutting resistance. A smaller G* means a softer material with better flowability and construction workability.

As shown in Figure 16, which focuses on the effect of secondary polymerization products on the rutting factor and fatigue factor of asphalt, the three-dimensional network secondary polymerization products differ from alcoholysis products (which exert a plasticizing effect) and aminolysis products (which enhance hydrogen bonds). Instead, they mainly rely on spatial structural support to significantly improve the high-temperature rutting resistance of asphalt. These products form a continuous, stable physical cross-linking network within the asphalt matrix, which effectively fixes the asphaltene colloidal structure and inhibits high-temperature flow and deformation, thereby increasing the asphalt modulus and rutting factor. Additionally, the three-dimensional network structure can disperse stress, delay the initiation and propagation of cracks, and simultaneously improve the fatigue factor of asphalt [26,27].

The rutting factor G*/sinδ is adopted to evaluate high-temperature rutting resistance, and the fatigue factor G*sinδ is used to assess fatigue performance (δ is the phase angle). Generally, G* decreases with rising temperature; asphalt softens at high temperatures and exhibits reduced deformation resistance. The variation in G* after modifier incorporation reflects the modification effect on the network structure of asphalt.

As illustrated in Figure 17, which depicts the effect of secondary polymerization products on the elastic recovery of asphalt at 5 °C and 25 °C, these products effectively improve the asphalt’s elastic recovery rate under low-temperature conditions. Specifically, the flexible aliphatic chains and ether bond structures in the secondary polymerization product molecules help maintain the flexibility of the asphalt colloid and relieve the low-temperature shrinkage stress. Meanwhile, the network cross-linked structure of the products avoids the low-temperature brittleness of asphalt. This comprehensive effect achieves performance coordination—restricting high-temperature softening deformation and suppressing low-temperature brittleness—thus meeting the technical requirements for high-modulus and high-viscosity asphalt. Such modified asphalt is therefore suitable for harsh engineering application scenarios, including high-grade pavements, heavy-load pavements, and long-life pavements.

### 3.3. Brief Summary

The ester and polyether segments in alcoholysis products form an amphiphilic structure, which plays plasticizing, viscosity-reducing, and phase-compatibilizing roles in asphalt, and forms a stable adsorption layer at the asphalt-aggregate interface, significantly improving water stability, making it suitable as a warm-mix agent or anti-stripping agent. Secondary-polymerized products form a three-dimensional physically cross-linked network within the asphalt matrix, which effectively immobilizes the asphaltene colloidal structure and suppresses high-temperature flow and deformation. Consequently, the modulus, high-temperature rutting resistance and fatigue resistance of asphalt are significantly improved.

## 4. Conclusions

This work investigates the high-value recycling of waste PET for the fabrication of sustainable asphalt additives. Two chemical routes, alcoholysis and secondary polymerization, were comparatively analyzed to clarify the structure–performance relationship and modification mechanism of PET-derived products. The results show that homogeneous alcoholysis at 220 °C achieves efficient and controllable PET degradation, yielding amphiphilic products suitable as warm-mix and anti-stripping agents [28]. By comparison, repolymerized products with three-dimensional cross-linked networks serve as high-modulus and high-viscosity modifiers. The amphiphilic ester groups reduce asphalt viscosity and enhance water resistance, whereas the cross-linked network remarkably improves high-temperature rutting resistance. This chemical modification strategy effectively addresses the poor compatibility and limited modification performance associated with conventional physically blended PET-modified asphalt. Future research will introduce aminolysis routes and high-efficiency catalysts coupled with continuous production techniques to optimize degradation efficiency, product purity and manufacturing stability. ^1^H-NMR or hydroxyl-value titration will be adopted to quantitatively characterize terminal-hydroxyl content of alcoholysis oligomers to obtain accurate stoichiometric ratio for secondary polymerization. Molecular simulations and interfacial characterization including SEM and Cole–Cole rheological analysis will be employed to elucidate the microscale interaction and phase-separation mechanism among modifiers, asphalt binders, and aggregates. Additionally, long-term aging, fatigue resistance, water stability, and life-cycle assessment of modified mixtures will be evaluated. Further engineering promotion will focus on the application of PET-based additives in recycled mixtures, cold-laid pavements, ultra-thin overlays, and high-altitude regions, aiming to expand the industrial applicability of such eco-friendly modifiers.

## Figures and Tables

**Figure 1:**
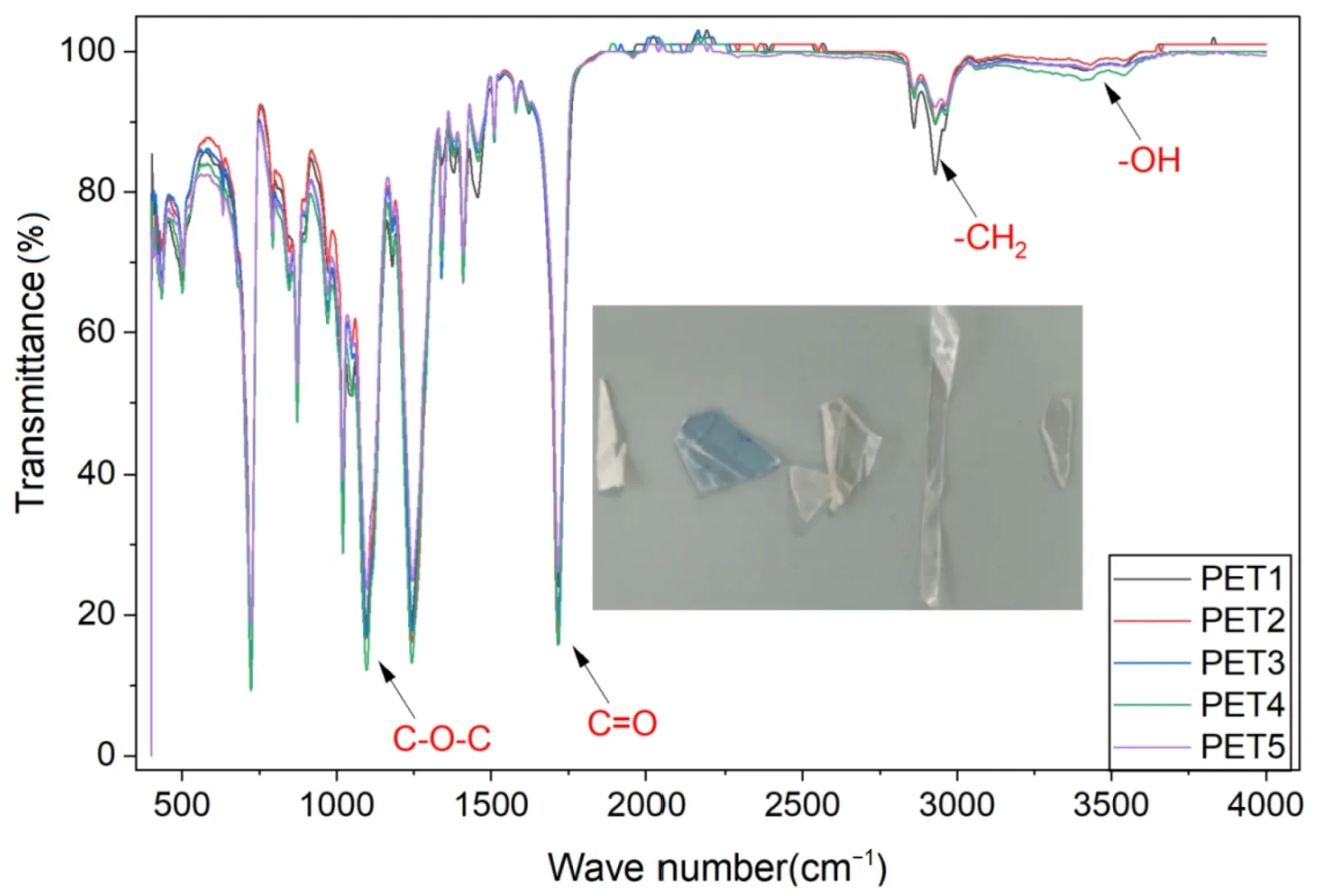
FTIR spectra of different waste PET samples. (PET1−PET5 are only for raw-material characterization and are not used for degradation reactions).

**Figure 2:**
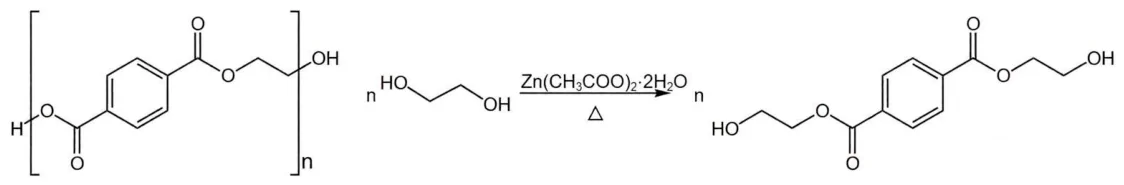
Alcoholysis reaction schemes of waste PET with ethylene glycol (EG).

**Figure 3:**
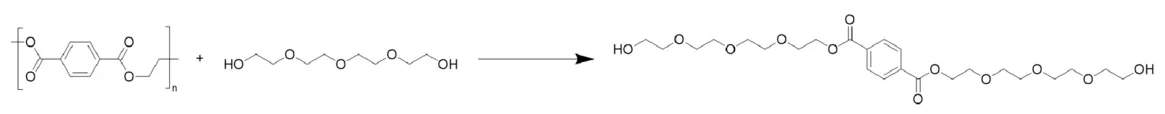
Alcoholysis reaction schemes of waste PET with polyethylene glycol (PEG).

**Figure 4:**
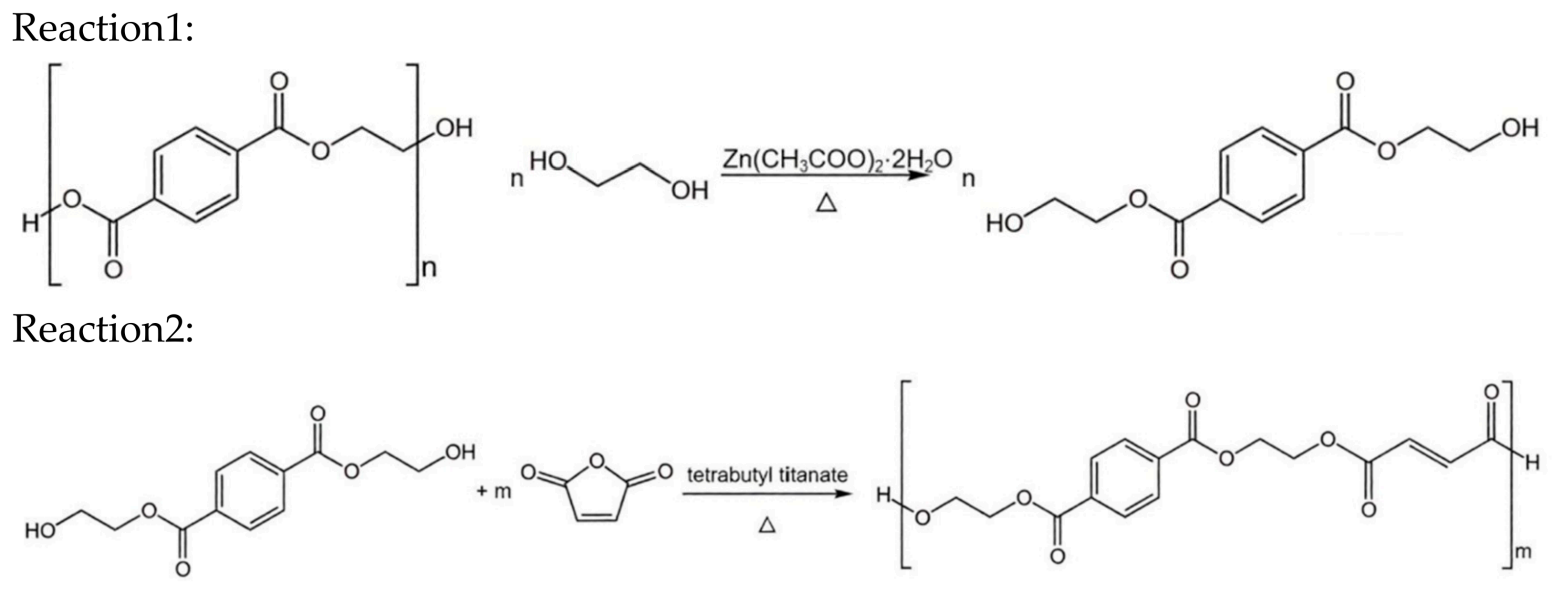
Secondary polymerization pathways of PET degradation products with glutaric anhydride (GAH) as an example.

**Figure 5:**
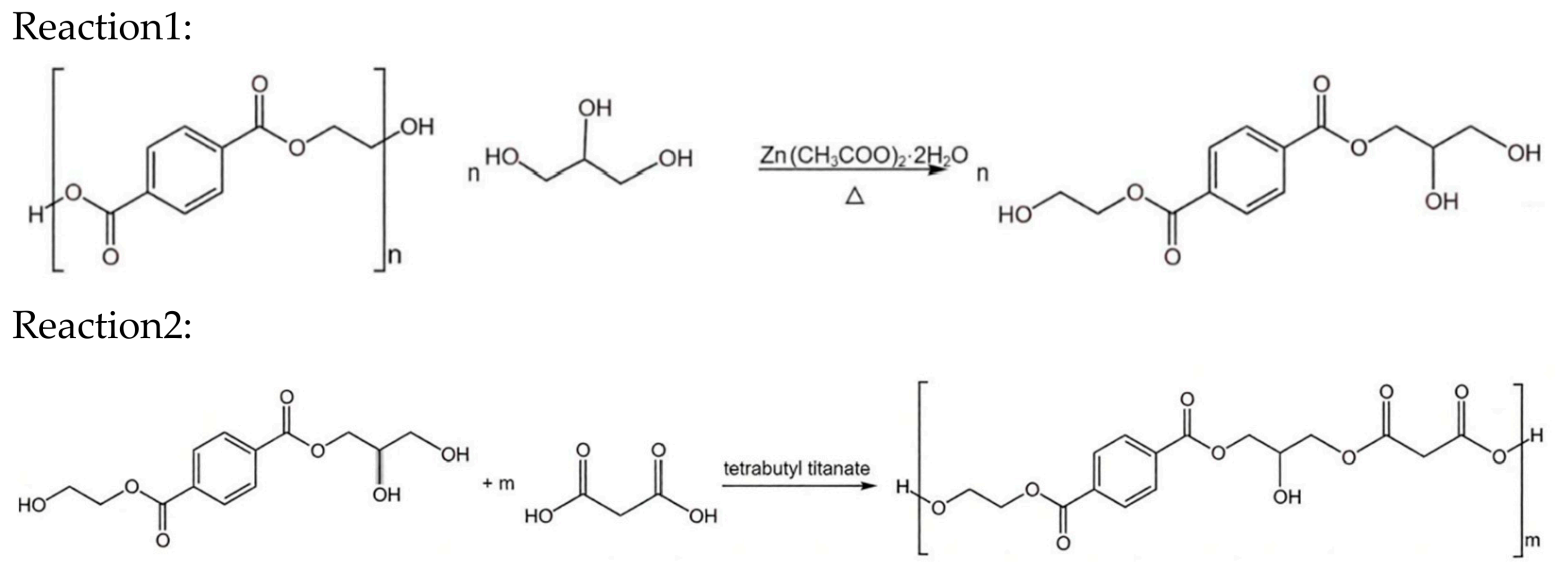
Secondary polymerization pathways of PET degradation products with malonic acid (MA) as an example.

**Figure 6:**
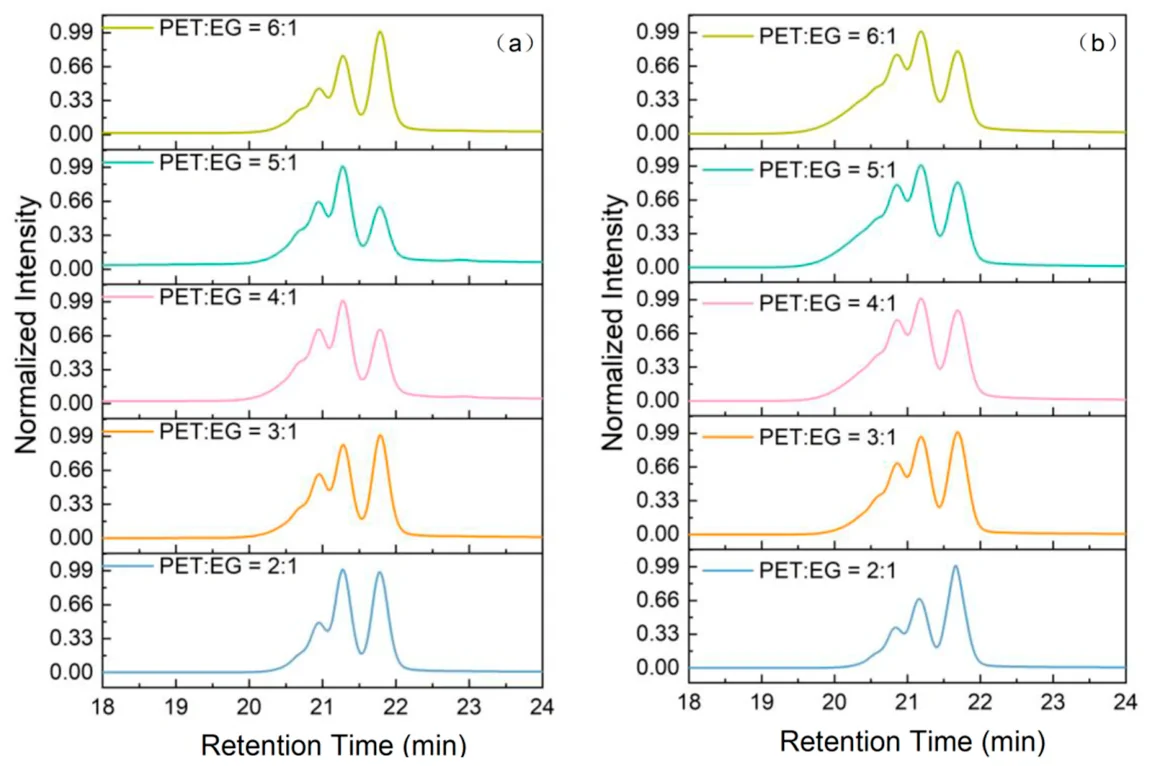
Molecular-weight distributions after alcoholysis at 160 °C (**a**) and 220 °C (**b**) with different PET/EG ratios.

**Figure 7:**
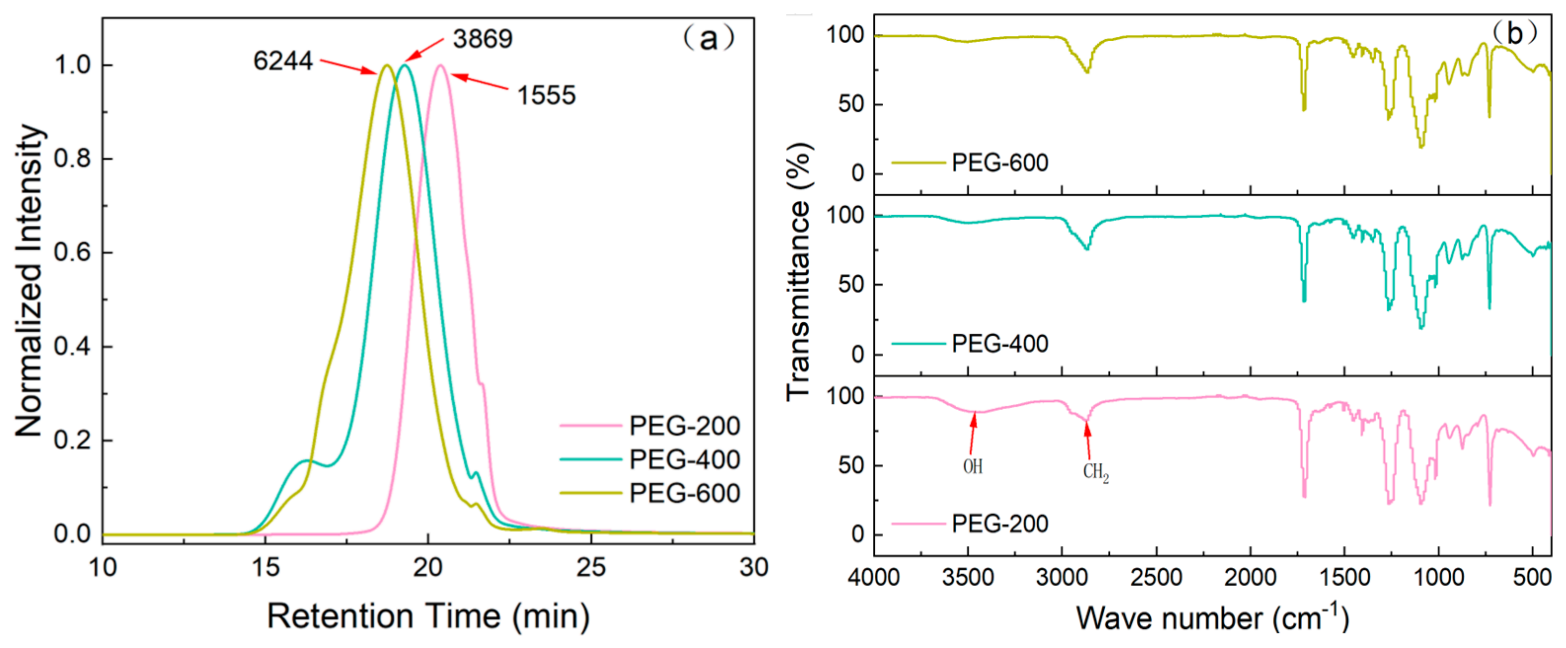
Molecular-weight normalization (**a**) and FTIR of PET after alcoholysis with PEG-200/400/600 (**b**).

**Figure 8:**
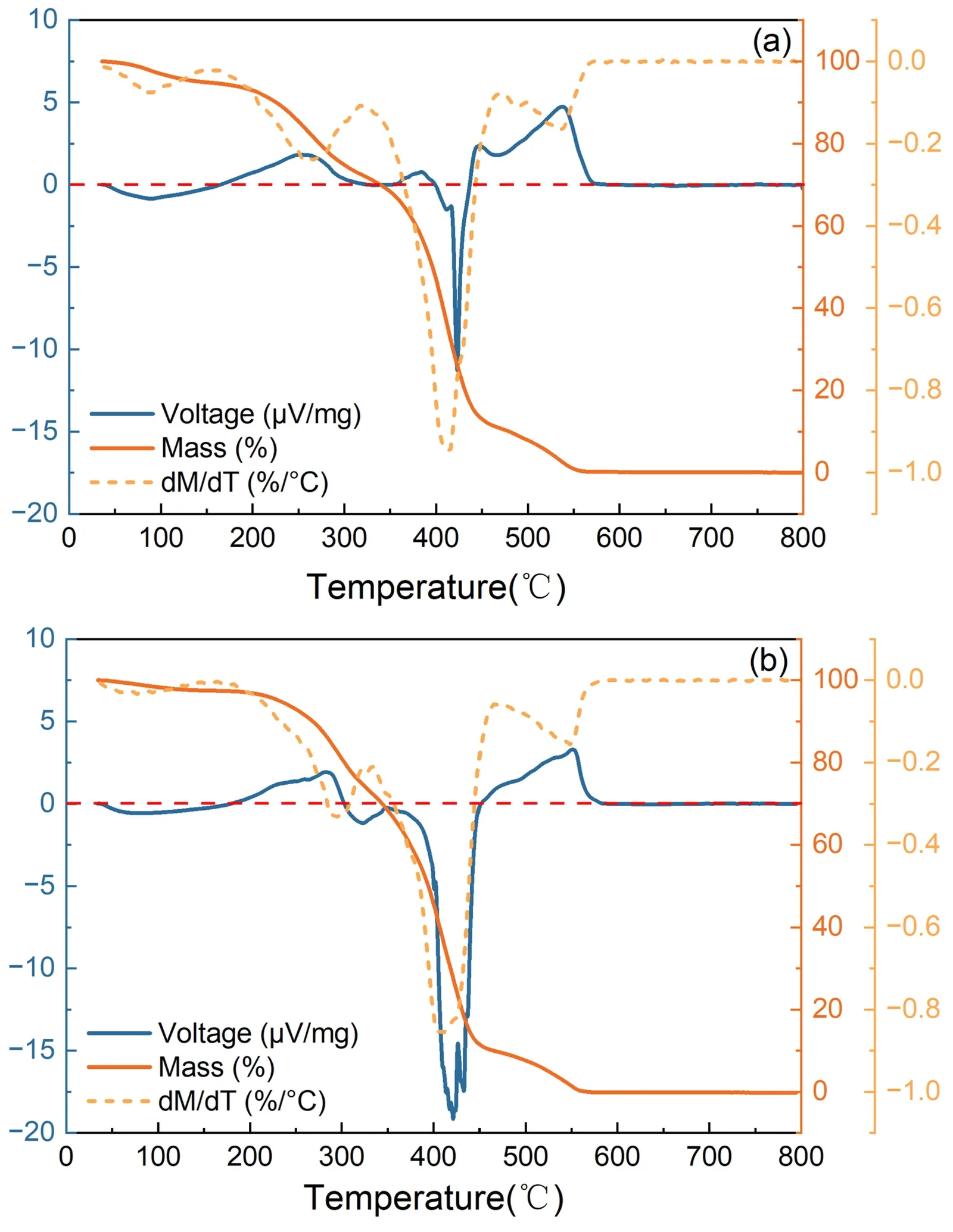

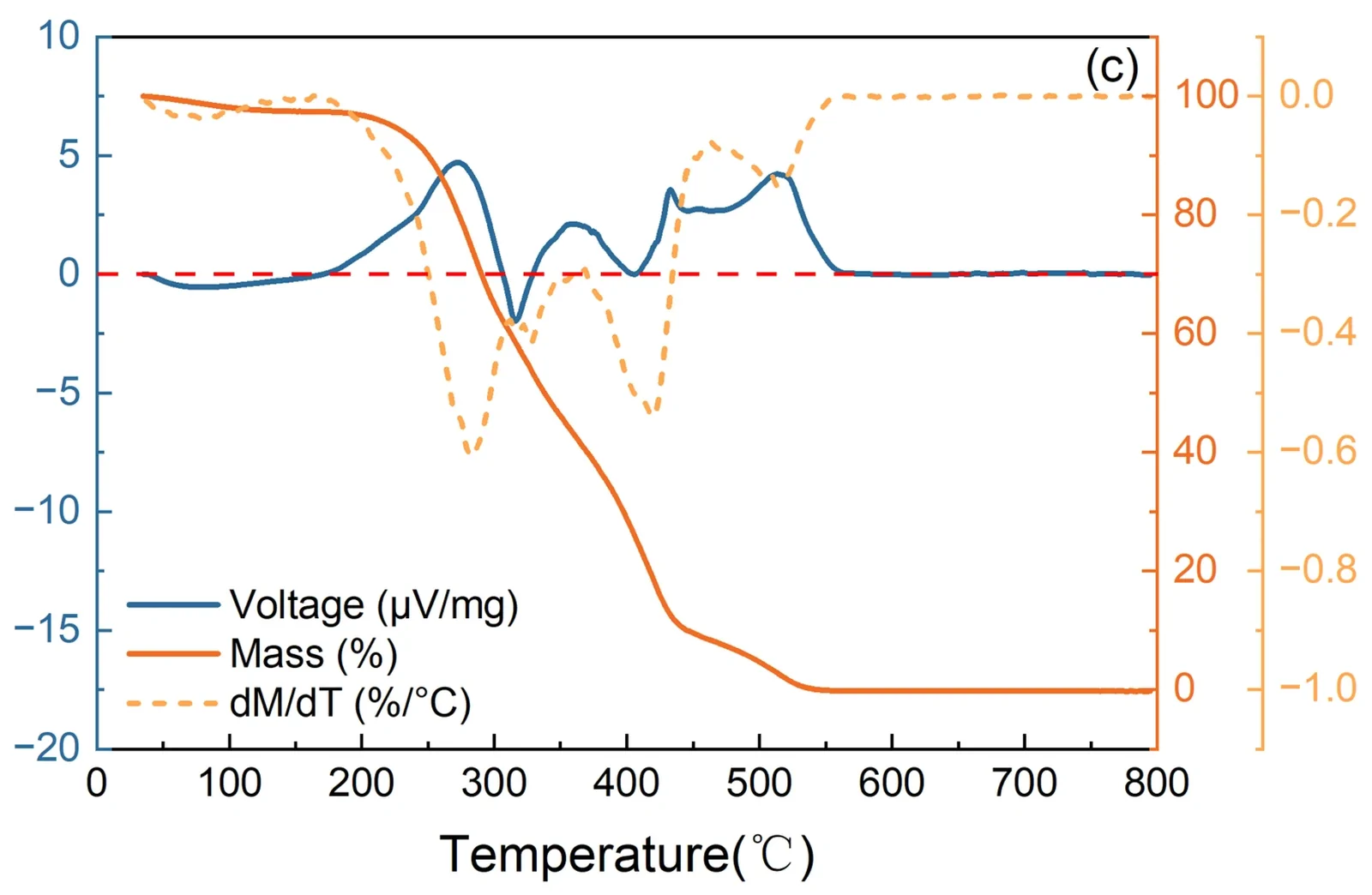
TGA-DTA curves of PEG-200 (**a**)/PEG-400 (**b**)/PEG-600 (**c**) alcoholysis products.

**Figure 9:**
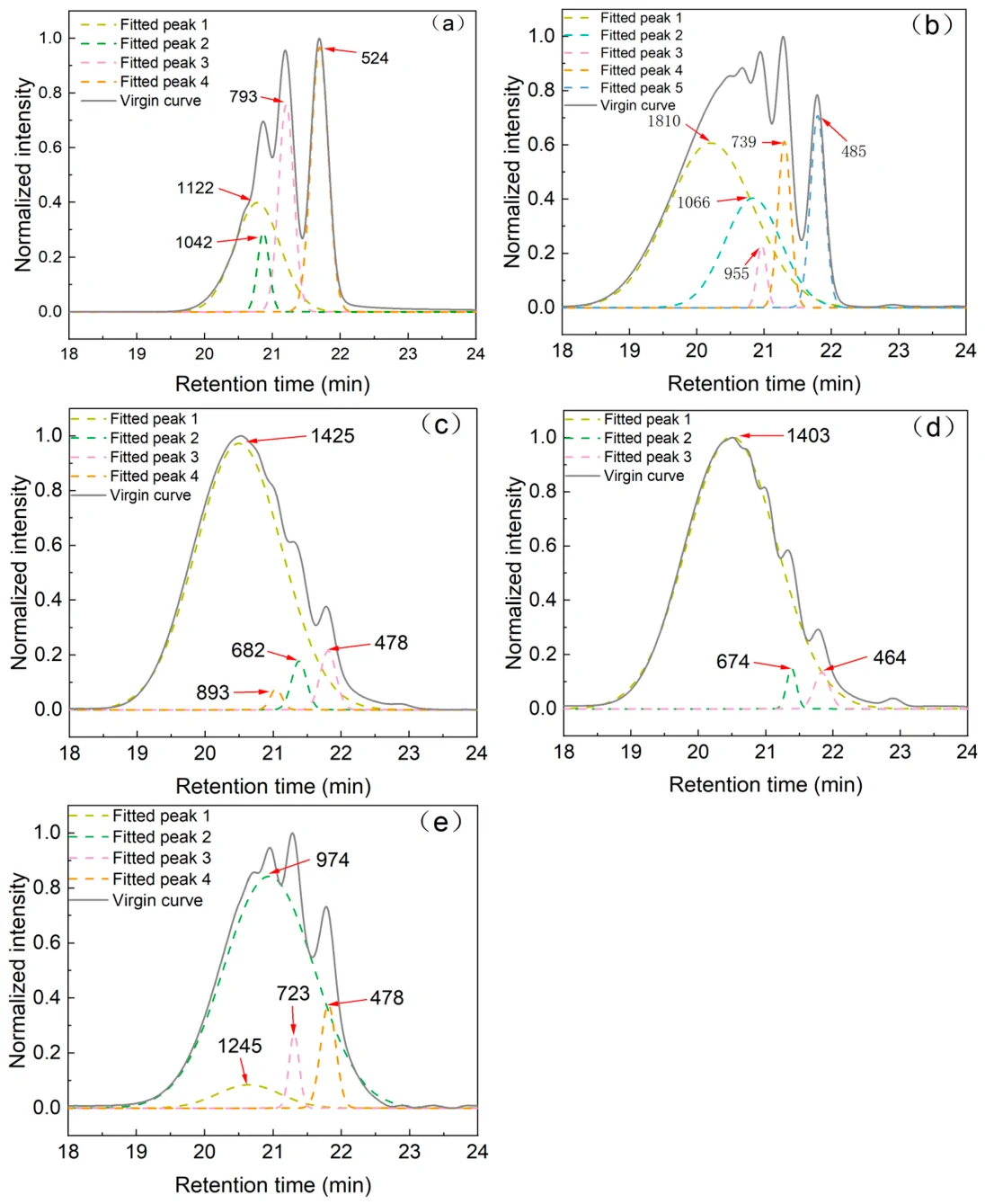
Molecular-weight normalization of secondary polymerization products based on degraded PET/EG alcoholysis (**a**), MA (**b**), SAA (**c**), GAH (**d**), TMA (**e**).

**Figure 10:**
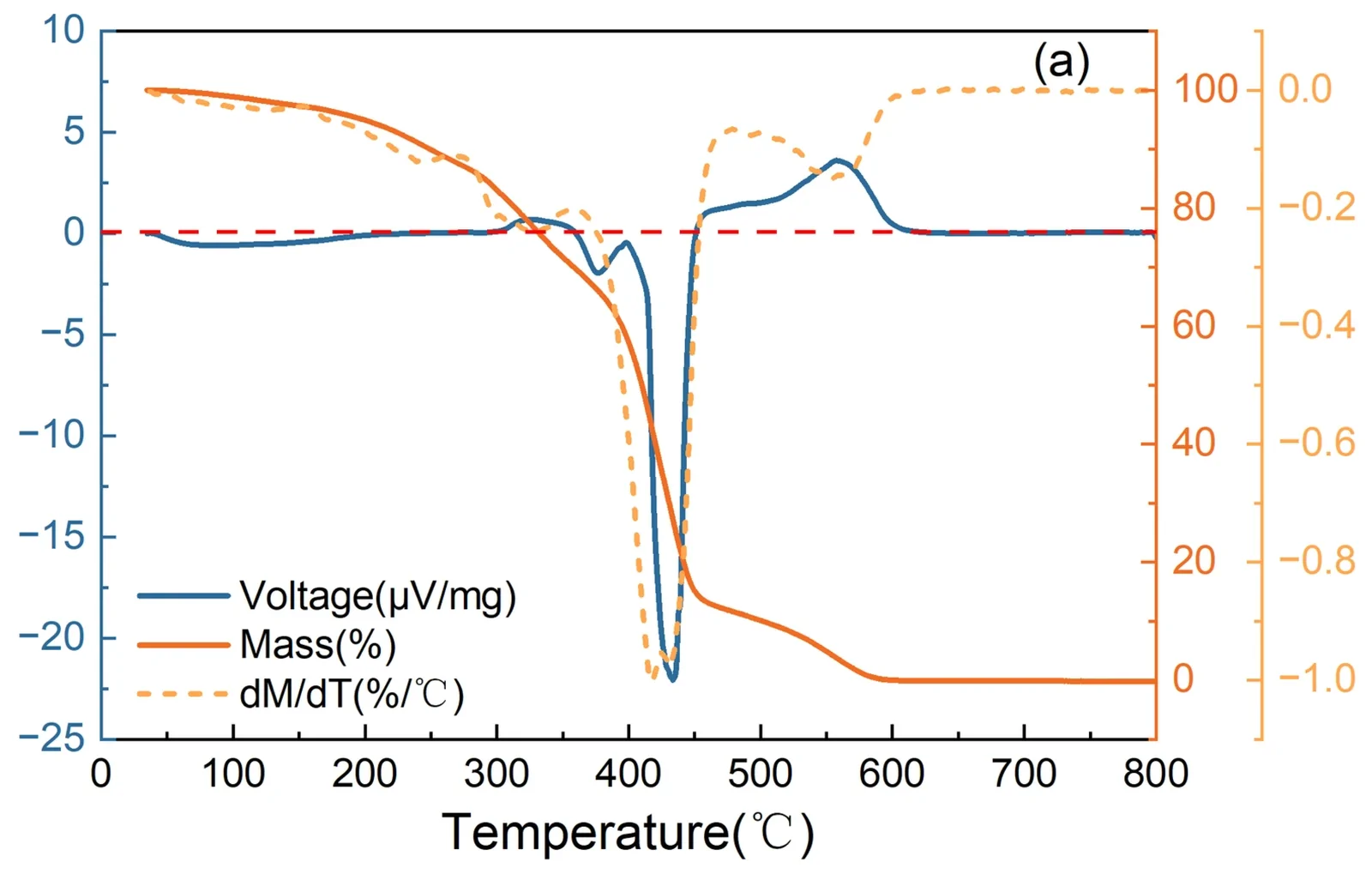

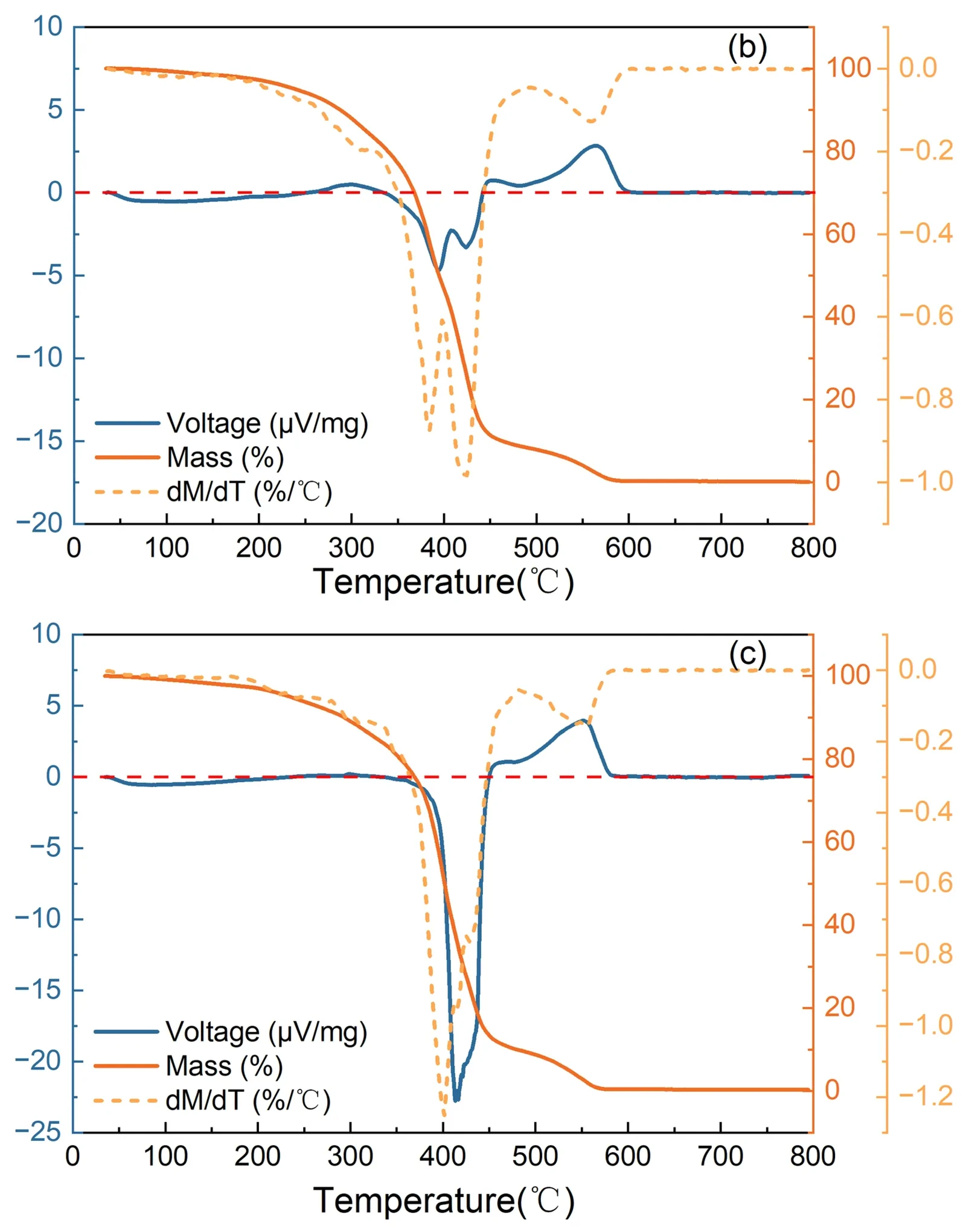

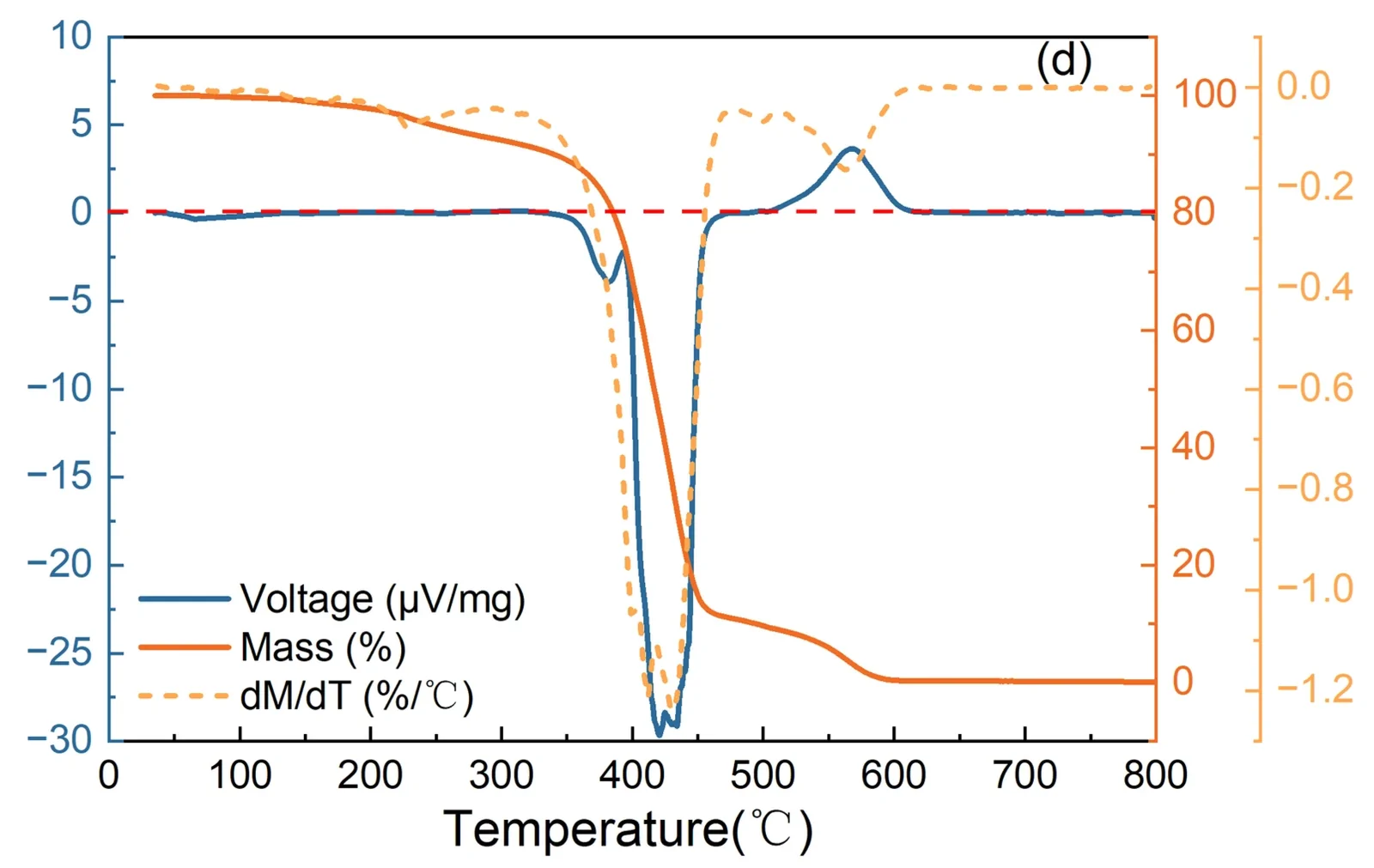
TGA-DTA curves of secondary polymerization products MA (**a**), SAA (**b**), GAH (**c**), TMA (**d**).

**Figure 11:**
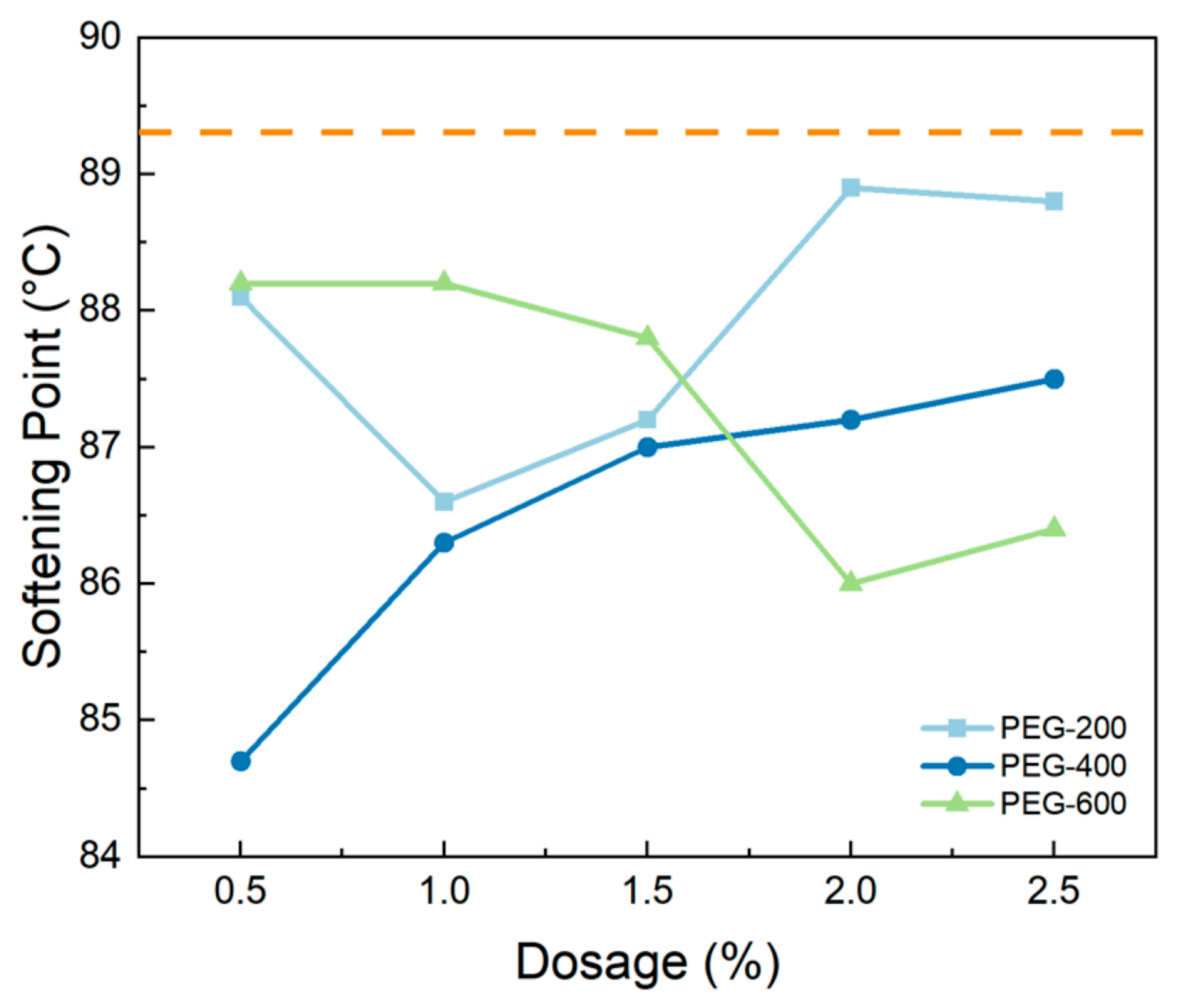
Softening point of 4.5 wt% SBS-modified asphalt with different dosages of alcoholysis-series PET additives.

**Figure 12:**
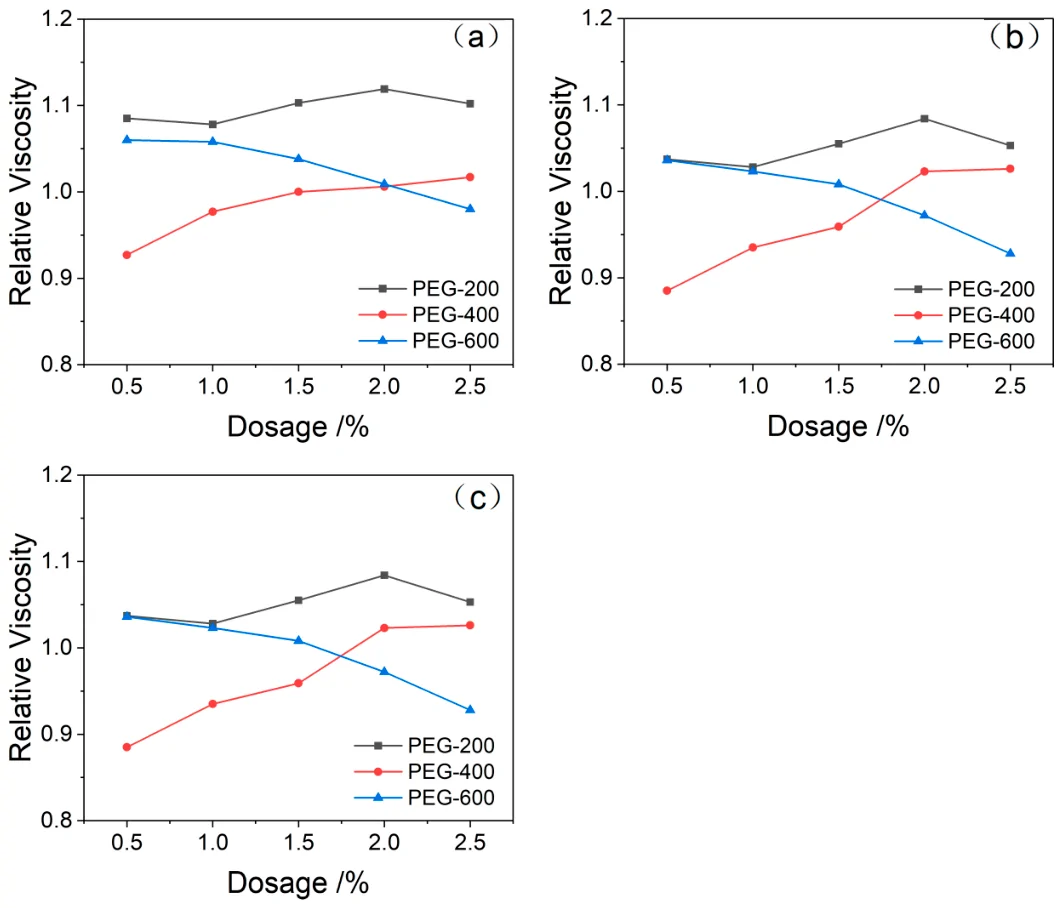
Viscosity of 4.5 wt% SBS-modified asphalt blended with PET alcoholysis additives at different temperatures (135 °C (**a**); 150 °C (**b**); 180 °C (**c**)). Brookfield rotational viscosity was measured at a rotational speed of 150 r/min (approximately 20 s^−1^ shear rate).

**Figure 13:**
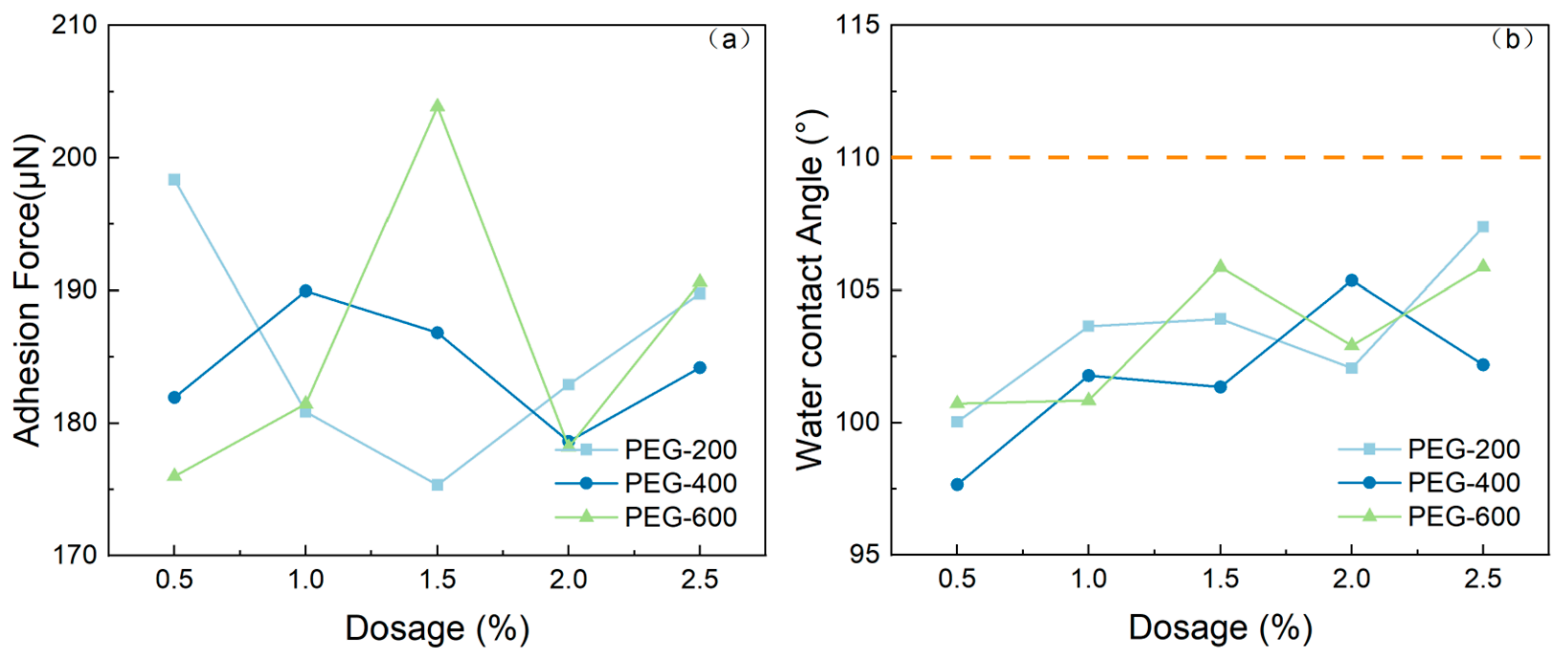
Effect of PEG alcoholysis products on adhesion force (**a**) and water contact angle (**b**) of 4.5 wt% SBS-modified asphalt.

**Figure 14:**
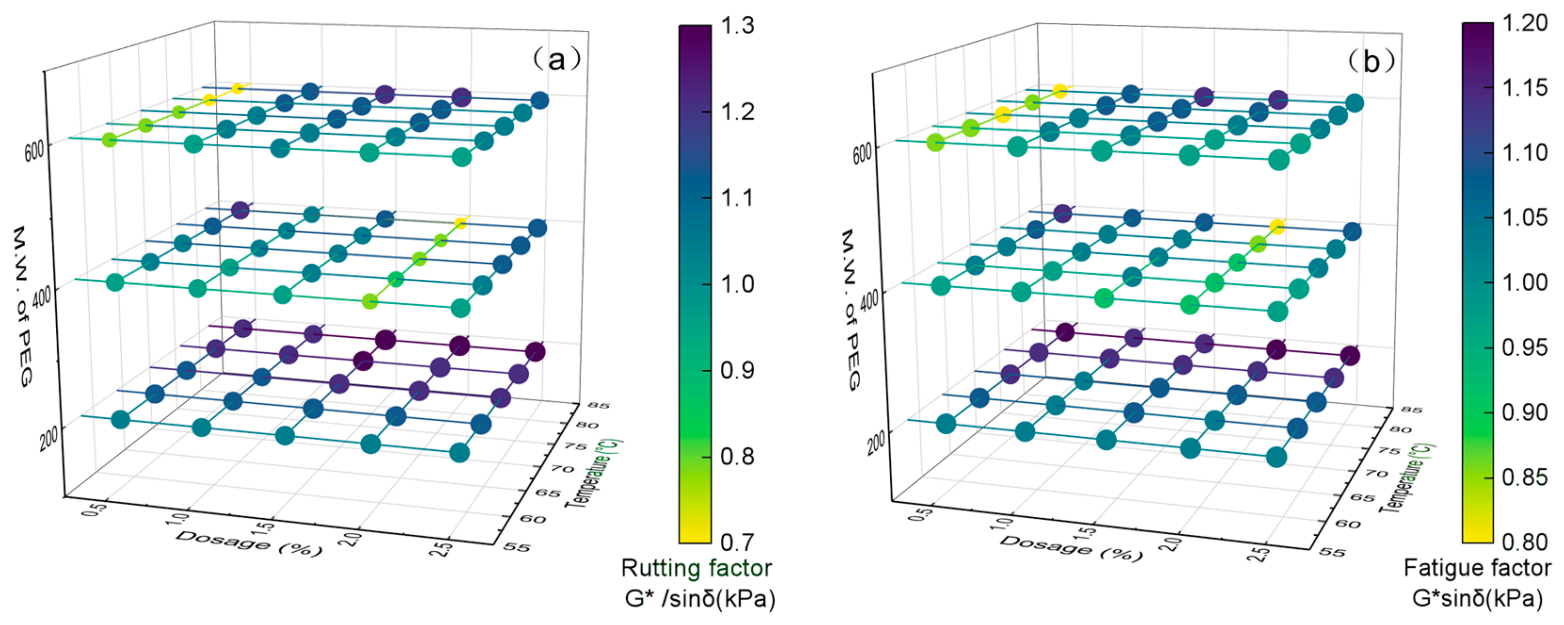
Effect of PEG alcoholysis products on rutting factor (**a**) and fatigue factor (**b**) of 4.5 wt% SBS-modified asphalt.

**Figure 15:**
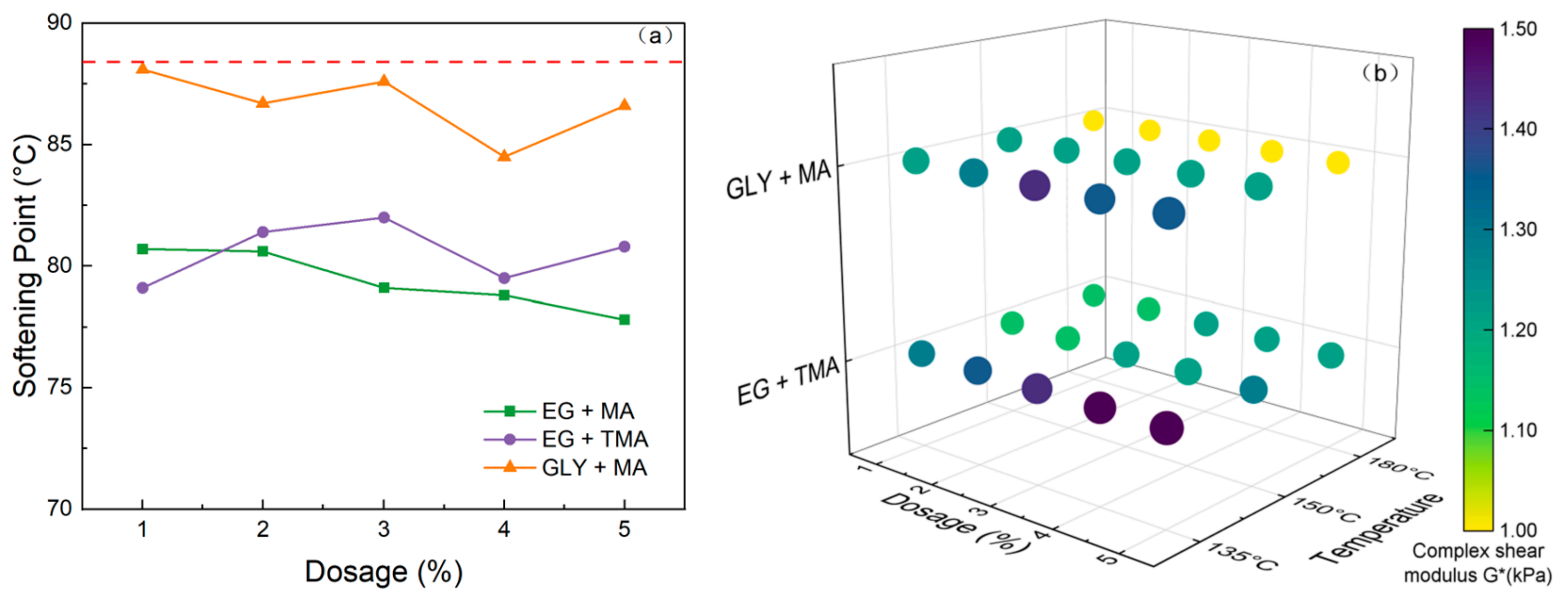
Effect of secondary polymerization products on softening point (**a**) and complex shear modulus G* (**b**) of ZH90 base asphalt.

**Figure 16:**
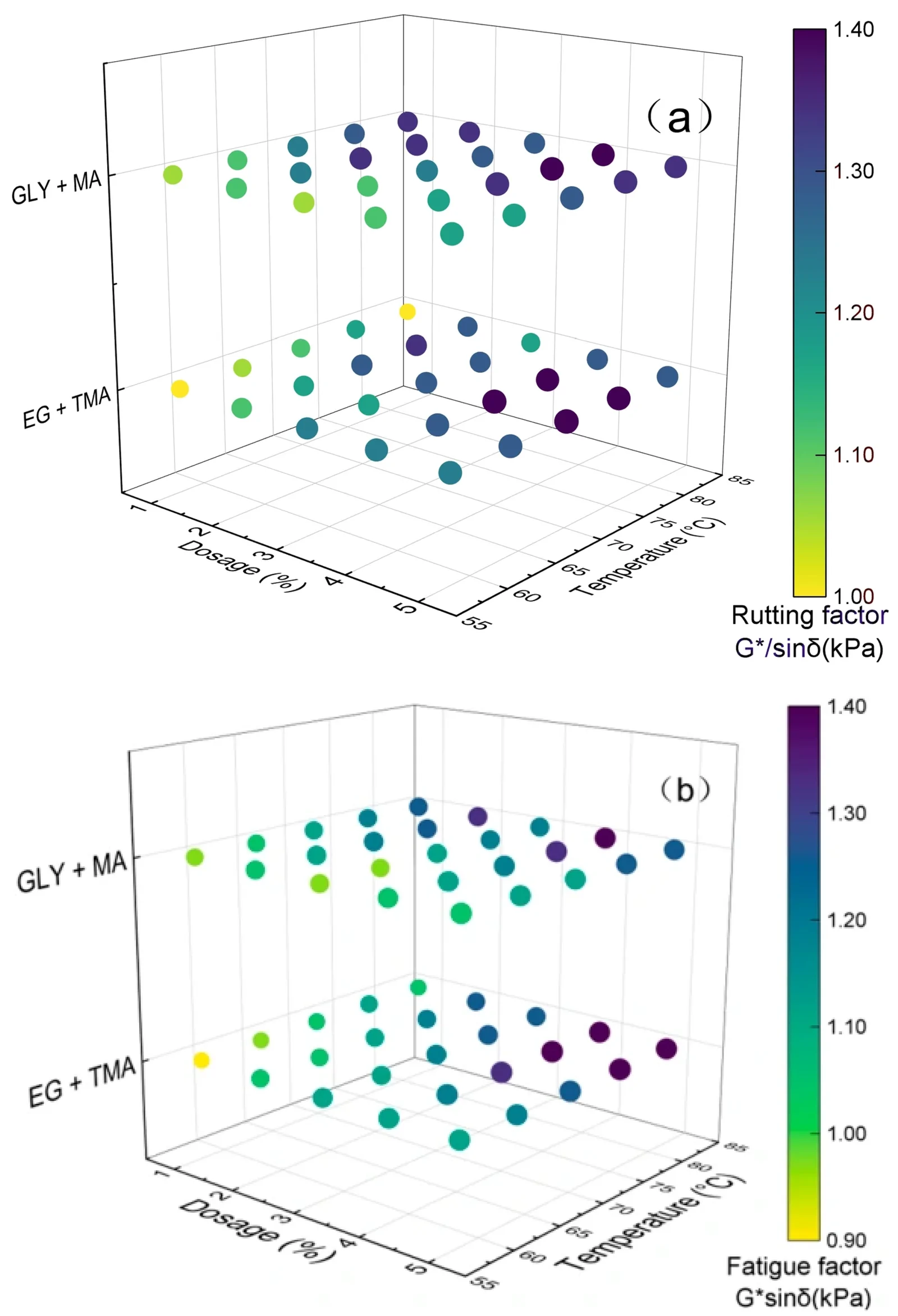
Effect of secondary polymerization products on rutting factor (**a**) and fatigue factor (**b**) of ZH90 base asphalt.

**Figure 17:**
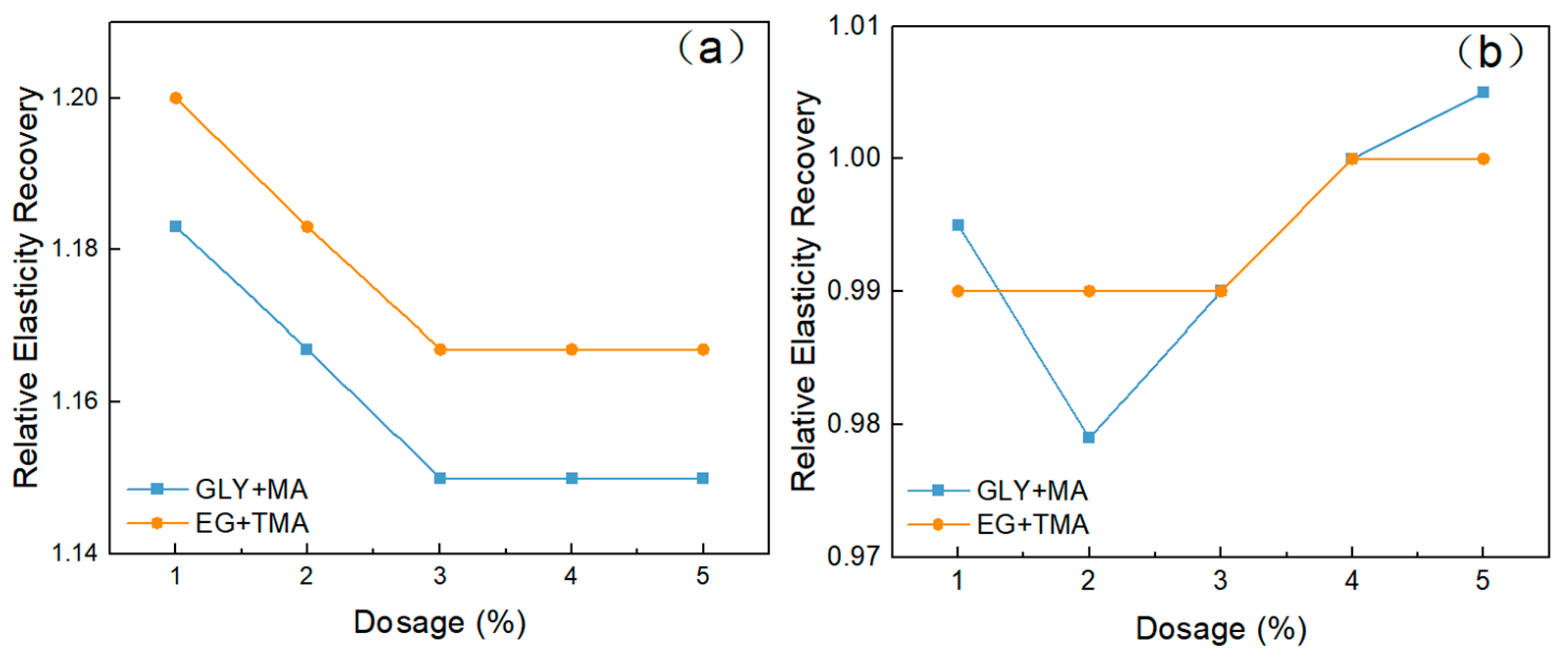
Effect of secondary polymerization products on elastic recovery of ZH90 base asphalt at 5 °C (**a**) and 25 °C (**b**).

**Table 1:** Mixing proportions of PET-derived additives for asphalt modification (mass percentage relative to asphalt binder mass).

Modifier Series	Additive Dosage				
Alcoholysis-series modifiers	0.5%	1%	1.5%	2%	2.5%
Repolymerization-series modifiers	1%	2%	3%	4%	5%

## Data Availability

The original contributions presented in this study are included in the article. Further inquiries can be directed to the corresponding authors.
